# Early Enoxaparin Treatment in a Newborn with Cerebral Venous Sinus Thrombosis and Acute Cerebellar Hemorrhage

**DOI:** 10.1055/a-2566-3952

**Published:** 2025-04-21

**Authors:** Margherita Velardi, Rita Luciano, Simonetta Costa, Mirta Corsello, Tommaso Verdolotti, Luca Massimi, Domenico M. Romeo, Francesca Gallini, Giovanni Vento

**Affiliations:** 1 Department of General Pediatrics and Pediatric Emergency, Bambino Gesù Children's Hospital, Istituto di Ricerca e Cura a Carattere Scientifico, Rome, Italy; 2 Unit of Neonatology, Department of Woman and Child Health and Public Health, Catholic University of Sacred Heart, Fondazione Policlinico Universitario A. Gemelli IRCCS, Rome, Italy; 3 Institute of Radiology, Fondazione Policlinico Universitario “A. Gemelli,” IRCCS, Rome, Italy; 4 Pediatric Neurosurgery Unit, Catholic University of Sacred Heart, Fondazione Policlinico Universitario A. Gemelli IRCCS, Rome, Italy; 5 Paediatric Neurology Unit, Catholic University of Sacred Heart, Fondazione Policlinico Universitario A. Gemelli IRCCS, Rome, Italy

**Keywords:** neonatal intracranial hemorrhage, neonatal cerebral thrombosis, anticoagulant therapy, cerebral sinus venous thrombosis, treatment timing

## Abstract

**Objective:**

To evaluate the management of anticoagulant therapy in neonates with cerebral sinus venous thrombosis (CSVT), focusing on potential benefits and risks.

**Study Design:**

We report the case of a full-term neonate diagnosed with CSVT, highlighting the rationale for early anticoagulation with unfractionated heparin. A brief literature review supports our clinical decision-making, considering current evidence and expert consensus despite limited neonatal-specific guidelines.

**Results:**

Heparin therapy was started shortly after diagnosis, without hemorrhagic complications. Neuroimaging showed complete thrombus resolution. The neonate had a normal neurological examination at discharge. Follow-up confirmed overall good clinical condition and showed mild axial hypotonia and convergent strabismus suggestive of cortical visual impairment.

**Conclusion:**

Management of CSVT in neonates remains debated. While heparin may carry a risk of bleeding, delaying treatment can lead to thrombus progression. Our case supports the potential safety and efficacy of early heparin use in selected patients. Tailored, risk-based decisions may improve outcomes, though further studies are needed to establish standardized protocols.

## Introduction

Cerebral sinus venous thrombosis (CSVT) is a focal or diffuse interruption of cerebral blood flow, resulting from occlusion of venous sinuses or cerebral veins, with or without intraparenchymal hemorrhage. Despite being a rare event in the neonatal period, with an estimated annual incidence of around seven cases per 1 million neonates, 1 CSVT represents a pathological condition of increasing interest in the neonatal field. 2 The wide spectrum of onset clinical manifestations makes it difficult to obtain an early diagnosis. Moreover, this condition is complicated by high morbidity and mortality in the neonatal period and there is still an open debate regarding the management of anticoagulant therapy and its timing. 3 4 Low molecular weight heparin in patients with CSVT may cause an intraparenchymal hemorrhage or may worsen it when already present at diagnosis, while a watchful wait with a supportive treatment may result in thrombus propagation and consequently additional brain damage. 4 5

We present a neonate with CSVT who has been successfully treated with heparin at an early time, despite the presence at diagnosis of a cerebellar hemorrhage. Parental informed consent was taken to publish the case.

## Case Report

A 39 ^1/7^ weeks of gestation male neonate weighting 3,200 g, was admitted to our neonatal intensive care unit (NICU) at 13 hours of life because of neurological symptoms such as difficulty in sucking, hypotonia, hyperflexion of the upper limbs and extension of the lower limbs, associated with mild respiratory distress and hyperglycemia.

He was born from a pregnancy obtained by homologous intracytoplasmic sperm injection (ICSI). The maternal TORCH complex serologies, the vaginal and straight swabs were negative and no signs of chorioamnionitis were reported. Fetal ultrasound scans (US) performed during pregnancy were reported as normal. Maternal coagulation screenings were negative. The mother had received therapy with acetylsalicylic acid during pregnancy and levothyroxine as a therapy for gestational hypothyroidism.

The baby was born from an urgent cesarean section performed because of a pathologic cardiotocographic pattern (type II) and a meconium-stained liquor. The newborn did not require any respiratory assistance at birth and the APGAR score was 9 both at 1 and 5 minutes. The arterial cord gas analysis showed a mild metabolic acidosis (pH: 7.08; BE: −11.2), the modified Sarnat score was normal, and then no indications to proceed to hypothermic treatment were identified. The newborn was admitted to the nursery for cardiorespiratory and glycemic monitoring and subsequently to the rooming-in ward after a clinical re-evaluation resulted as normal. At 13 hours of life, neurological symptoms such as difficulty in sucking, hypotonia, hyperflexion of the upper limbs, and extension of the lower limbs, associated with mild respiratory distress and hyperglycemia were observed.

**Fig. 1:**
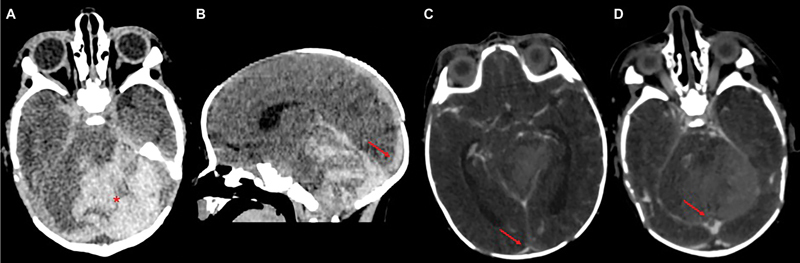
( **A, B** ) Noncontrast enhanced CT scan. ( **C, D** ) CT angiography scan. CT scan showed acute hemorrhage located in the left cerebellar hemisphere (asterisk in A), exerting mass effect (IV ventricle compression, upward transtentorial herniation, and crowding of foramen magnum were noted). A high density of posterior superior sagittal sinus was noted (arrows in B). CTA showed multiple filling defects, representing thrombosis, in the posterior superior sagittal sinus (“empty delta” sign, arrow in C) and Erofilo's torcularis region (arrow in D).

The brain US performed at the admission to the NICU, showed a hyperechoic lesion in the posterior cranial fossa, extending in the median area above the cerebellum. This finding was consistent with cerebellar hemorrhage. Due to the term gestational age and the clinical history, a diagnosis of CSVT was hypothesized and an emergency cerebral computed tomography angiography was performed at 20 hours of life. The exam confirmed the presence of an intraparenchymal acute hemorrhage located in the left cerebellar hemisphere, with upward transtentorial herniation and compression on the brainstem and fourth ventricle. The lesion was associated with thrombosis of the Erofilo's Torcularis and the posterior part of the superior sagittal sinus and incomplete thrombosis of the left transverse sinus and straight sinus ( Fig. 1 ). The newborn started anti-convulsant therapy with barbiturates after onset of apneic crisis requiring noninvasive respiratory assistance. The electroencephalogram showed a symmetrical low-voltage electrical activity with sporadic anomalies on the posterior regions, especially on the left side.

**Fig. 2:**
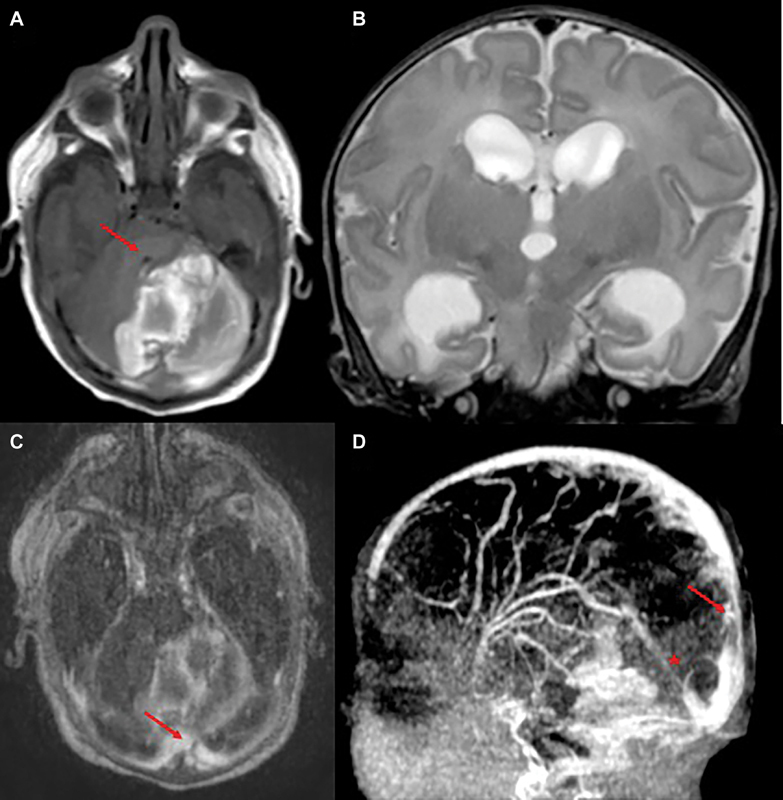
( **A** ) Spin echo (SE) T1 weighted sequence, axial plane. ( **B** ) Turbo SE (TSE) T2 weighted sequence, coronal plane. ( **C** ) Magnetic resonance venography (MRV), axial plane ( **D** ) MRV, and MIP reconstruction. MR showed left cerebellar hemorrhage compressing the IV ventricle (arrow in A) and causing ventricular dilatation (B). MRV showed a filling defect in the left transverse sinus (arrow in C) and reduced lumen of the posterior sagittal sinus (arrow in D) and of the rectus sinus (asterisk in d), as for partial recanalization.

**Fig. 3:**
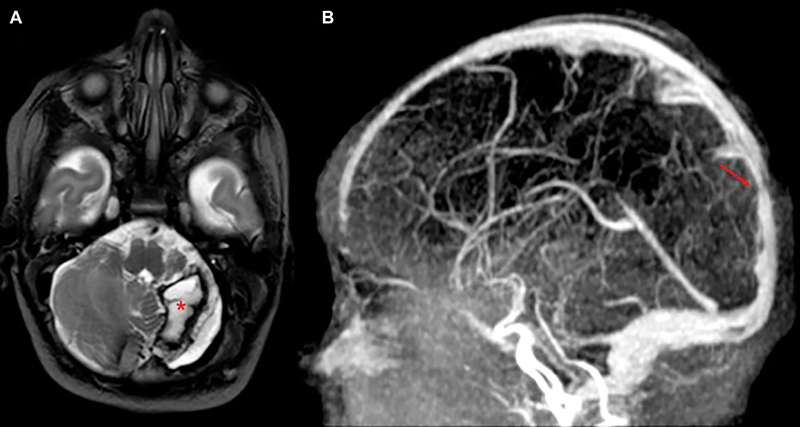
( **A** ) TSE T2 weighted sequence, axial plane. ( **B** ) MRV, MIP reconstruction. MR showed the chronic phase of the hemorrhage. A hemosiderin-lined cavity was detected in the left cerebellar (asterisk in A). Reduced expansion of the left posterior cranial fossa was noted. No signs of vascular malformation were detected. MRV showed focal reduction of the lumen of the posterior sagittal sinus.

Early treatment with low molecular weight heparin at a prophylaxis dosage (75 IU/kg every 12 hours) was started at 48 hours of life to prevent the propagation of thrombosis. The cerebral US performed before starting the treatment had already documented the stability of the hemorrhage. Due to the consistent risk of hemorrhage expansion at this early stage, brain US monitoring was planned every 8 hours to check the evolution of the hemorrhage. The serial brain US showed a stable hemorrhage and mild ventricular dilation (lateral and third ventricles). The anticoagulant therapy was progressively increased in relation to the anti-Xa factor monitoring, up to a therapeutic dose of 155 IU/kg every 12 hours. Cerebral magnetic resonance imaging (MRI) performed on the seventh day of life confirmed the size stability of the cerebellar hemorrhage and showed partial recanalization of the Erofilo's torcularis, with the persistence of small filling defect in the transverse sinus ( Fig. 2 ). According to neurological stability and absence of apneic crisis, respiratory assistance was discontinued on the 14th day of life. Predischarge cerebral US showed a cavitated lesion in the site of the cerebellar hemorrhage and a stable mild ventriculomegaly. The newborn was discharged on the 23rd day of life in good general health on heparin therapy and barbiturates. A scheduled follow-up clinic was planned including brain US, neurosurgical and neurological evaluation, and blood tests. At 45 days of life, the follow-up brain US showed an almost complete regression of the bleeding. The brain MRI performed at this stage documented the expected regression of the hemorrhagic lesion in the posterior cranial fossa, the decompression of the brainstem, the resolution of the herniation, and the reduction of the supratentorial ventricular dilation. Brain MRI also showed a residual focal thinning of the Silvio aqueduct and an irregular appearance of the posterior portion of the superior sagittal sinus and of the mesial portion of the left transverse sinus. The remaining dural venous sinuses and internal cerebral veins were patent ( Fig. 3 ).

**Fig. 4:**
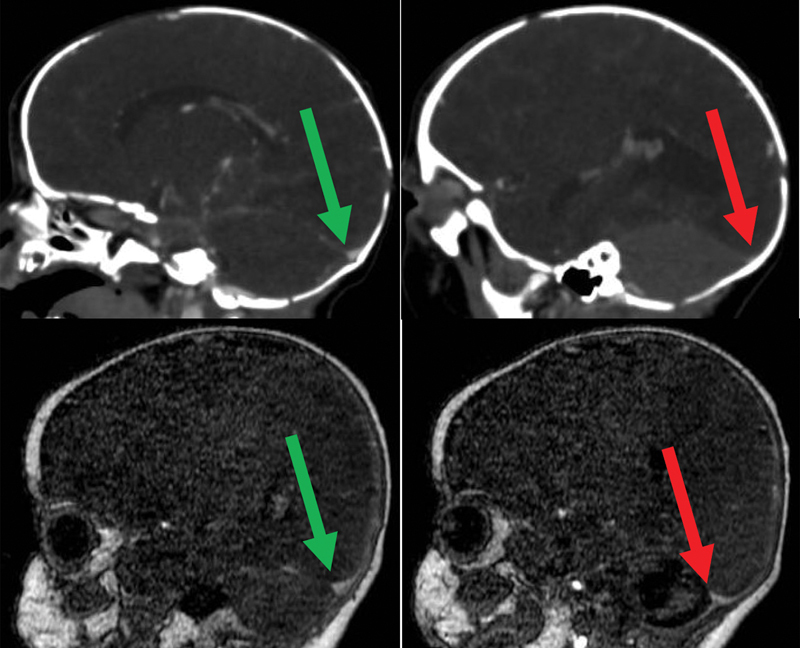
First row shows the computed tomography angiography at diagnosis: the green arrow indicates patent right transverse sinus and the red arrow indicates nonopacified proximal left transverse sinus. The second row shows the contrast-enhanced magnetic resonance angiography at follow-up: the green arrow shows the right patent transverse sinus and the red arrow shows the left patent transverse sinus.

The brain MRI performed at age 7 months documented a complete recanalization of Erofilo's torcularis and transverse sinus ( Fig. 4 ). As a consequence, the anticoagulant therapy was discontinued. At the 10-month follow-up control, the child was found in good general health and showed normal weight, length, and head circumference. The neurologic assessment performed at the same age showed mild axial hypotonia and convergent strabismus related to a cortical visual impairment.

## Discussion

The clinical history of the reported case was suggestive of CSVT, thus allowing a prompt neuro-radiological diagnosis. In fact, the intraparenchymal hemorrhage, the symptoms observed, and the clinical features identified, such as medically assisted pregnancy, gestational age at term, urgent cesarean section, and mild fetal distress, were coherent with the hypothesis of CSVT. Once the CSVT diagnosis was confirmed, the therapeutic management was not univocal. The currently available literature regarding neonates with CSVT does not include RCTs and it is not conclusive about therapeutic management. In the case of intraparenchymal hemorrhage associated with CSVT, current guidelines, and protocols suggest watchful waiting and supportive therapy with re-evaluation of MRI at 5 to 7 days. 6 7 8 9 However, the currently available data show more favorable outcome rates (39–50%) 10 and lower thrombus propagation frequencies (3%) 11 in infants undergoing early anticoagulation therapy (within 72 hours of diagnosis) compared with untreated infants (20% favorable outcome and 25% thrombus propagation, respectively 12 ). Intraparenchymal hemorrhages already present at the time of CSVT diagnosis have been reported in 30 to 80% of cases. 13 Noteworthy, bleedings related to anticoagulant therapy have been reported in only 8% of treated cases while thrombus propagation has been associated with a high risk of new venous infarction (40%). 13

The duration of anticoagulant therapy is also controversial. The British Committee for Standards in Haematology stated that children with CSVT and no intraparenchymal hemorrhage should receive anticoagulant therapy with heparin, continued for a minimum of 3 months in cases of reversible provoking illness, and for 6 months in the absence of provoking illness, and for longer periods of time in patients with a long-lasting or genetic risk factor or with persistent symptomatic venous outflow obstruction. Moreover, it is recommended that repeated imaging should be considered prior to stopping anticoagulant therapy. 8 The European Pediatric Neurology Society guidelines on the anticoagulant treatment of CSVT in children and neonates stated that half of the recurrences of thrombosis occur within 3 months, and two-thirds within 6 months, after the initial accident. For this reason, a 3 to 6-month period has been proposed as the standard duration of anticoagulation in most studies, although the duration of treatment should be adapted to each situation. 14 On the other hand, the American College of Chest Physicians stated that for neonates with CSVT without significant intraparenchymal hemorrhage, anticoagulation is suggested for a total duration between 6 weeks and 3 months. 7 Faced with this controversy over the duration of treatment, we chose to be cautious with our patient and to extend the anticoagulant therapy until we had documented the complete recanalization of the venous sinuses by neuroimaging.

Multicenter RCT studies are needed to decide the optimal management of this condition. In the meanwhile, the discussion of this clinical case is an occasion to re-open a debate regarding the efficacy, safety, and duration of enoxaparin early administration in neonates with CSVT and active hemorrhage on the basis of the recent encouraging results reported in the literature.

## References

[1] Martinelli I Bucciarelli P Passamonti S M Battaglioli T Previtali E Mannucci P M Long-term evaluation of the risk of recurrence after cerebral sinus-venous thrombosis Circulation 2010 121 25 2740 2746 20547928 10.1161/CIRCULATIONAHA.109.927046

[2] Fitzgerald K C Williams L S Garg B P Carvalho K S Golomb M R Cerebral sinovenous thrombosis in the neonate Arch Neurol 2006 63 03 405 409 16533968 10.1001/archneur.63.3.405

[3] Bhatt M D Chan A K Venous thrombosis in neonates Fac Rev 2021 10 20 33718937 10.12703/r/10-20 PMC7946391

[4] Kouzmitcheva E Moharir M Neonatal cerebral sinovenous thrombosis: the anticoagulation debate Dev Med Child Neurol 2018 60 09 853 29675894 10.1111/dmcn.13785

[5] Rossor T Arichi T Bhate S Hart A R Raman Singh R Anticoagulation in the management of neonatal cerebral sinovenous thrombosis: a systematic review and meta-analysis Dev Med Child Neurol 2018 60 09 884 891 29675941 10.1111/dmcn.13760

[6] American Heart Association Stroke Council Council on Cardiovascular Disease in the Young Roach E S Golomb M R Adams R Management of stroke in infants and children: a scientific statement from a Special Writing Group of the American Heart Association Stroke Council and the Council on Cardiovascular Disease in the Young Stroke 2008 39 09 2644 2691 18635845 10.1161/STROKEAHA.108.189696

[7] Monagle P Chan A KC Goldenberg N A Antithrombotic therapy in neonates and children: antithrombotic therapy and prevention of thrombosis, 9th: American College of Chest Physicians evidence-based clinical practice guidelines Chest 2014 146 06 1694 10.1378/chest.11-2308 PMC3278066 22315277

[8] British Committee for Standards in Haematology Chalmers E Ganesen V Liesner R Guideline on the investigation, management and prevention of venous thrombosis in children Br J Haematol 2011 154 02 196 207 21595646 10.1111/j.1365-2141.2010.08543.x

[9] Yang J Y Chan A K Callen D J Paes B A Neonatal cerebral sinovenous thrombosis: sifting the evidence for a diagnostic plan and treatment strategy Pediatrics 2010 126 03 e693 e700 20696732 10.1542/peds.2010-1035

[10] Berfelo F J Kersbergen K J van Ommen C H Neonatal cerebral sinovenous thrombosis from symptom to outcome Stroke 2010 41 07 1382 1388 20522810 10.1161/STROKEAHA.110.583542

[11] Moharir M D Shroff M Stephens D Anticoagulants in pediatric cerebral sinovenous thrombosis: a safety and outcome study Ann Neurol 2010 67 05 590 599 20437556 10.1002/ana.21936

[12] Ramenghi L A Cardiello V Rossi A Neonatal cerebral sinovenous thrombosis Handb Clin Neurol 2019 162 267 280 31324314 10.1016/B978-0-444-64029-1.00012-6

[13] Moharir M D Shroff M Pontigon A M A prospective outcome study of neonatal cerebral sinovenous thrombosis J Child Neurol 2011 26 09 1137 1144 21628696 10.1177/0883073811408094 PMC3695693

[14] French Society for Paediatric Neurology European Paediatric Neurology Society Lebas A Chabrier S Fluss J EPNS/SFNP guideline on the anticoagulant treatment of cerebral sinovenous thrombosis in children and neonates Eur J Paediatr Neurol 2012 16 03 219 228 22425391 10.1016/j.ejpn.2012.02.005

